# Discontinuation of antidepressant medication in primary care supported by monitoring plus mindfulness-based cognitive therapy versus monitoring alone: design and protocol of a cluster randomized controlled trial

**DOI:** 10.1186/s12875-019-0989-5

**Published:** 2019-07-26

**Authors:** Carolien Wentink, Marloes J. Huijbers, Peter Lucassen, Cornelis Kramers, Reinier Akkermans, Eddy Adang, Jan Spijker, Anne E. M. Speckens

**Affiliations:** 1 Department of Psychiatry, Radboud University Medical Center, Postbus 9101, 6500 HB Nijmegen, the Netherlands; 2 Department of Primary and Community Care, Radboud University Medical Center, Postbus 9101, 6500 HB Nijmegen, the Netherlands; 3 Department of Pharmacology and Toxicology and Department of Internal Medicine, Radboud University Medical Center, Postbus 9101, 6500 HB Nijmegen, the Netherlands; 4 Department for Health Evidence, Radboud University Medical Center Nijmegen, Postbus 9101, 6500 HB Nijmegen, The Netherlands; 5 Pro Persona Nijmegen, GGZ, Reinier Postlaan 6, 6525 GC Nijmegen, The Netherlands

**Keywords:** Antidepressants, Discontinuation, Tapering, Long-term use, General practitioner, Primary health care, Mindfulness-based cognitive therapy

## Abstract

**Background:**

Antidepressant use continues to rise, mainly explained by an increase in the proportion of patients receiving long term treatment. Although treatment guidelines recommend discontinuation after sustained remission, discontinuing antidepressants appears to be challenging for both patients and general practitioners (GPs). Mindfulness-Based Cognitive Therapy (MBCT) is an effective intervention that reduces the risk of relapse in recurrent depression and might facilitate discontinuation by teaching patients to cope with withdrawal symptoms and fear of relapse. The current study aims to investigate the effectiveness of the combination of Supported Protocolized Discontinuation (SPD) and MBCT in comparison with SPD alone in successful discontinuation of long-term use of antidepressants in primary care.

**Methods:**

This study involves a cluster-randomized controlled trial conducted in primary care patients with long-term use antidepressants with baseline and 6, 9 and 12 months follow-up assessments. Patients choosing to discontinue their medication will be offered a combination of SPD and MBCT or SPD alone. Our primary outcome will be full discontinuation of antidepressant medication (= 0 mg) within 6 months after baseline assessment. Secondary outcome measures will be the severity of withdrawal symptoms, symptoms of depression and anxiety, psychological well-being, quality of life and medical and societal costs.

**Discussion:**

In theory, stopping antidepressant medication seems straightforward. In practice however, patients and their GPs appear reluctant to initiate and accomplish this process. Both patients and professionals are in need of appropriate tools and information to better support the process of discontinuing antidepressant medication.

**Trial registration:**

ClinicalTrials.gov PRS ID: NCT03361514 retrospectively registered October 2017.

## Background

Antidepressant use continues to rise. Between 2003 and 2013, antidepressant prescription numbers have doubled in western countries [1], and have been rising with 3% per year in the Netherlands [2]. Despite being effective for some patients in reducing symptoms of depression and anxiety and reducing the risk of depressive relapse [3] antidepressants may also have side effects such as sleep disturbance, weight gain, sexual dysfunction and gastrointestinal bleeding [4, 5]. In addition, they sometimes even go along with more serious adverse events, such as falls, attempted suicide or self harm, stroke and epilepsy [6]. Consequently, current primary care guidelines advise to consider discontinuation of antidepressants 6 months after remission of a depression or 6–12 months after remission of an anxiety disorder. Patients with recurrent depression are advised to discontinue medication after 1–2 years. It is generally recommended to taper slowly, with guidelines suggesting a 50% dose reduction every 2 weeks while monitoring side effects and deploying relapse prevention strategies [7]. The pace of tapering seems important as it appears to influence the risk of relapse in patients with depressive disorder [8].

However, the steady rise of antidepressant use seems to be explained mainly by an increase in long-term users [9]. In the UK and US, 50–65% of patients treated with antidepressants continue to use them for more than 2 years [10–12]. After starting antidepressant medication almost 30% of the patients become chronic users, defined as the consecutive use of any antidepressant for at least 12 months [13]. Despite the general guidelines on tapering antidepressants in primary care, actually discussing and initiating discontinuation appears to be challenging [14, 15]. Stopping antidepressant medication might be hampered by the presence of physical or psychological withdrawal symptoms, including headache, dizziness, electric-shock sensations, sleep disturbance, anxiety and mood swings. These symptoms may easily be misidentified as signs of impending relapse [16]. In addition, earlier unsuccessful attempts to discontinue may provoke further fears for withdrawal symptoms and relapse. Previous research in a sample of 146 inappropriate long-term antidepressant users from 45 general practices in the Netherlands showed that a multidisciplinary, patient tailored advice to discontinue antidepressant medication was rejected in 48% of the cases. The proportion of successful antidepressant discontinuation following this multidisciplinary advice was only 6% [17].

To protect against depressive relapse after the acute phase of depression several psychotherapeutic treatment strategies have evolved [18]. This way alternatives for patients not wanting to maintain antidepressants seem available. Mindfulness Based Cognitive Therapy (MBCT) is one of these approaches of relapse prevention, it is an innovative psychological approach for relapse/recurrence prevention in recurrent depression developed by Segal, Williams & Teasdale [19]. It is an 8-week group-based treatment that integrates elements from mindfulness-based stress reduction (MBSR) [20] and cognitive–behavioural therapy (CBT) [21]. A recent meta-analysis showed that MBCT appears efficacious as a treatment for relapse prevention for those with recurrent depression. MBCT reduces the risk of depressive relapse/recurrence compared with the current mainstay approach, maintenance antidepressants [22]. As MBCT appears to be a viable alternative to antidepressant medication it is possible that mindfulness could provide patients support in the de-identification of thoughts and allowing of uncomfortable physical and mental symptoms, and also help them to deal with possible withdrawal effects.

There is some evidence that MBCT might be useful in supporting tapering of antidepressant medication. Two randomized controlled trials in the UK, offering recurrently depressed patients MBCT with additional tapering support, show successful tapering of Antidepressant Medication (ADM) in 75% [23] and 71% of participants [24]. However, results from a study conducted in the Netherlands were less promising. Only 53% of the patients allocated to the group who received MBCT and tapering support succeeded in tapering their antidepressants [25]. Remarkably, there was a relatively large number of “cross-overs” in this trial, as 12% of patients who were asked to continue their medication after MBCT actually discontinued their antidepressant medication successfully. This latter trial showed that tapering was not necessarily easy even after MBCT, but that the perspective of the patient (e.g. preferences, motives, previous experiences with withdrawal) may have played a significant role. The authors suggested that MBCT for tapering ADM might need to be offered in homogeneous groups of patients who are eager to withdraw from their medication and that it should be specifically tailored to address effects of discontinuation within the mindfulness framework.

Therefore, the aim of this study is to investigate the effectiveness of the combination of supported protocolized discontinuation (SPD) and mindfulness-based cognitive therapy (MBCT) in comparison with SPD alone in successfully discontinuing long-term use of antidepressants in primary care. SPD is carried out by the GP and mental health assistant (MHA). The function of the MHA (POH-GGZ in Dutch this is an abbreviation for Practice Support Professional for Mental Health Care) was installed in the Netherlands in 2008 to support GPs in diagnosing, treating and referring patients with mental health problems. In 2016 in the Netherlands 81% of all GP’s have arranged a MHA in their practice.

Our main hypothesis is that long-term users of antidepressants in primary care receiving SPD + MBCT will more often successfully discontinue their medication within a period of 6 months than patients receiving SPD alone. Our secondary hypotheses are that, in comparison with patients receiving SPD alone, patients who receive SPD + MBCT will have a lower rate of relapse/recurrence of a depressive or anxiety disorder, a lower rate of restarting medication, suffer less from withdrawal symptoms, report less depression, anxiety, and ruminative brooding, and more mindfulness skills, self-compassion and psychological wellbeing over the 12 months follow-up period.

## Methods

### Design

**Fig. 1:**
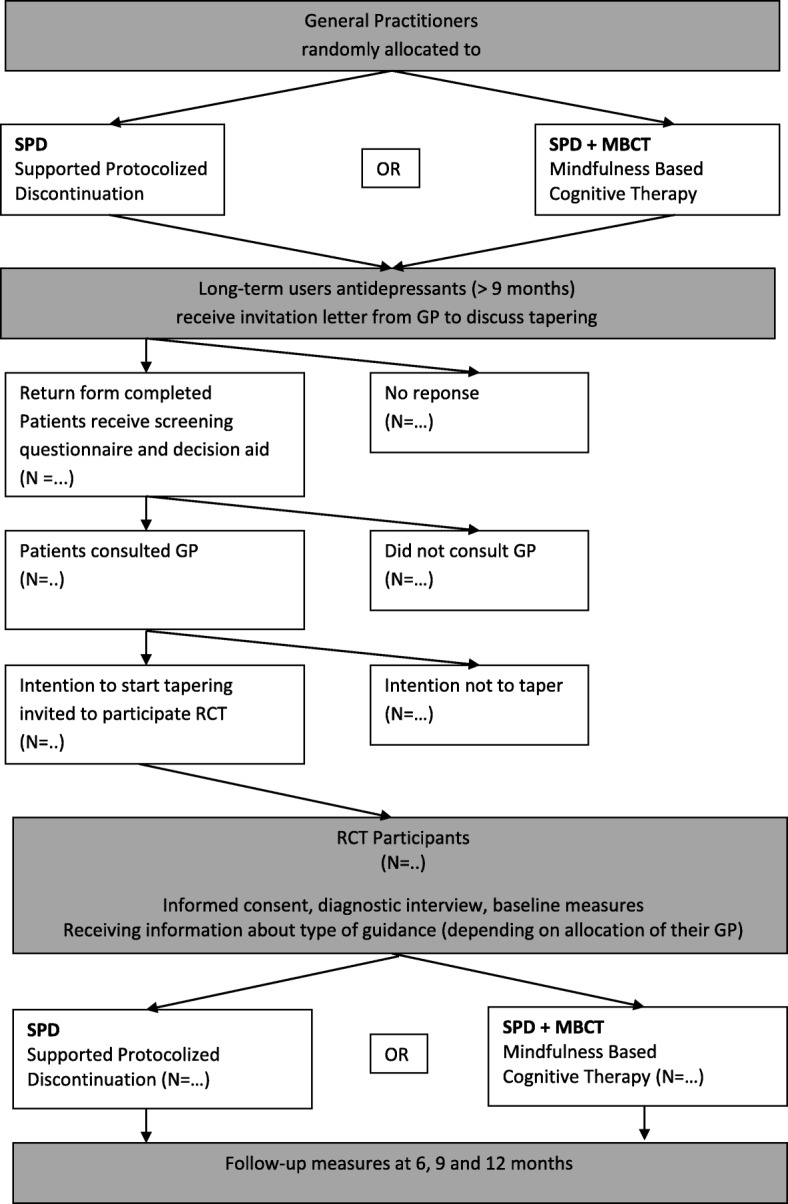
Flow chart of the recruitment and study procedure

This study is designed as a cluster-randomized controlled trial conducted in primary care with GP’s that work with a MHA, randomizing patients at the level of their GPs over a) Supported Protocolized Discontinuation (SPD) alone or b) SPD with additional Mindfulness-Based Cognitive Therapy (MBCT). We choose cluster randomisation for practical reasons, i.e. to simplify the procedures within the GP Practices. The patient, GP and the research team will be aware of the allocated condition. However, the mental health assistant MHA of the GP will be blind to the condition as much as possible by asking the GP not to inform the MHA about their allocated study arm. In addition we will ask participants in the SPD + MBCT condition to refrain from informing pro-actively about their participation in the MBCT. Regarding recruitment a detailed explanation is written in the Procedure. For a study flowchart see Fig. 1. The study protocol has been approved by the Medical Ethics Committee Arnhem-Nijmegen and registered under number 2016–2527.

### Participants

Patients will be recruited from GPs with a mental health assistant in the north, middle and east part of the Netherlands and only included in the study after informed consent has been obtained.

#### Inclusion criteria

In order to be eligible to participate in this study patients must have received prescriptions for antidepressants in primary care for at least the past 9 months and be 18 years or older.

#### Exclusion criteria

Patients who meet any of the following criteria will be excluded from the study: current treatment by a psychiatrist; current substance use disorder; non-psychiatric indication for long-term antidepressant usage (i.e. neuropathic pain); having participated in a mindfulness training (> 3 sessions) within the last 5 years; or inability to perform the assessments due to cognitive or language difficulties.

#### Safety and monitoring

Patients will be free to increase the dosage or restart their medication at any time during the study. In case of difficulties during the discontinuation process patients are advised to contact their GP and or MHA. Suspected serious adverse events will be recorded and reported to the Medical Ethics Committee Arnhem-Nijmegen.

### Sample size

Based upon earlier research we expect a discontinuation rate for patients in the SPD condition of 15% and a 40% discontinuation rate for patients in the SPD + MBCT condition. We used data from Eveleigh [17] to predict discontinuation in the SPD condition (where 6% successful discontinuation was observed according to very strict criteria, and we made adaptations to align with the context of the current setting, e.g. all patients had a clear intention to taper). To estimate the rate in the SPD + MBCT condition, we looked at studies with available data on adherence to tapering after MBCT, ranging between 53 and 71% [23–25]. Again we made a conservative adaptation of 40% to account for differences in time frame (6 versus 15 months), intention-to-treat versus per-protocol sample, and considering the difficulties in discontinuation and withdrawal symptoms as mentioned in the literature [16]. The following assumptions were used in the sample size calculation: power 80%, alpha of 5% and two tailed testing. Due to the fact that the trial will be cluster randomized, the calculated number of patients assuming individual randomization, has to be inflated by a design factor. To account for this an intra-class correlation (ICC) of 0.05 was used, which is commonly used for research including outcome measures [26]. With regard to the mentioned research we also assume a dropout rate of 15%. Taking these figures into account a total of 24 GPs have to be included, with 138 patients participating in the study; including 69 patients per described condition; presenting an estimate of 5 participating patients per GP.

### Procedure

Eligible GPs will be informed about the study and requested to participate. GP’s will be randomly allocated to either SPD alone or SPD + MBCT. The GPs are asked to identify patients using antidepressant medication for more than 9 months in their electronic prescription system. Between 2016 and 2018 interested GP’s will invite their long-term-users of ADM to discuss tapering via a letter and return form. GPs are requested to inform the research team about the number of patients who will not get an invitation including the reason(s).Patients’ reply forms will be collected by the research team and interested patients will be invited to complete the online screening questionnaire which includes demographic characteristics, ADM-use, the Patient Health Questionnaire [27] and Generalized Anxiety Disorder [28]. Subsequently, they will receive an information brochure about tapering, a decision aid and an invitation to make an appointment with their GP. Patient and GP are encouraged to use the decision aid as a conversation guideline to make a shared decision on whether or not to discontinue the antidepressant medication. After having consulted their GP about tapering and if they decide to start tapering, patients will receive an invitation to participate in the RCT and an invitation for a research interview. During the interview written informed consent that has been approved by the Medical Ethics Committee of Arnhem-Nijmegen will be obtained. The psychiatric structured diagnostic interview that will be conducted [29] will take about 60–90 min altogether. Patients will also be requested to complete baseline measures online. Thereafter patients will be advised to plan meetings with the Mental Health Assistant for their SPD. Those patients allocated to SPD + MBCT will be invited to participate in the first upcoming MBCT course. Follow up measurements take place six, nine and 12 months after baseline using telephone interviews and online questionnaires. The first assessment took place in February 2017, the last assessment will take place in July 2019.

### Interventions

#### Supported Protocolized discontinuation (SPD)

Patients who choose to discontinue their medication will make a personal tapering schedule with their GP based upon a discontinuation protocol with tapering suggestions. The protocol offers suggestions to taper within a maximum of 6 months describing all types of available dosages per ADM so individualized schedules can be constructed. The protocol is available upon request through the research team (CW). In addition, they will be offered supportive meetings with the GP’s mental health assistant. The assistant will receive basic information about discontinuation guidance, i.e. the information brochure, decision aid, discontinuation protocol and a short guideline how to organise consultations. The short guideline is available upon request through the research team (CW). Patients will be advised to discontinue their medication within 6 months.

#### Mindfulness based cognitive therapy (MBCT)

MBCT will be offered according to the treatment protocol developed for recurrent depression [30], adapted to the specific needs of patients discontinuing their antidepressant medication. The psycho-education sections about depression will be extended with information about pro and cons of stopping antidepressants, withdrawal effects and anticipatory anxiety. Based on our expectation that the discontinuation process may take longer than is currently regarded as common practice, sessions 1–4 will take place on a weekly and sessions 5–8 on a fortnightly basis. Each session will last 2.5 h with a 6-h silent day between session six and seven. In addition to the group sessions, participants will be instructed to practice mindfulness for approximately 30 min a day. Participants will receive a digital link to download guided meditations and yoga exercises for home practice. The mindfulness courses will be provided by teachers qualifying the advanced criteria of the Association of Mindfulness Based Teachers in the Netherlands and Flanders, which include a) relevant professional training; b) having attended MBSR / MBCT as a participant; c) a minimum of 150 h of education in MBSR/MBCT background and theory, training in teaching formal and informal meditation practices, psycho-education and inquiry, supervision and teaching an MBSR or MBCT course including a reflection report; d) teaching a minimum of two courses per 2 year; e) minimum of 3 years of practicing meditation regularly and attending retreats; and f) continued training. All teachers will receive additional training in using the specific study protocol at the start of the project. We will organize two peer supervision meeting per course during the intervention phase of the trial. Teaching sessions will be videotaped to check treatment integrity. A random selection of two tapes per teacher will be rated for treatment adherence and competency by highly experienced MBCT/MBSR teachers with the Mindfulness-Based Cognitive Therapy Adherence Scale [31].

### Outcome measures

Table 1 presents an overview of the outcome measures and the time points of assessments.

**Table 1:** Overview of the measures and corresponding time points

Measure	Target concept	Baseline (T0)	FU6 months (T1)	FU9 months (T2)	FU12 months (T3)
Daily calendar	Use of antidepressants	Daily registration during 12 months			
SCID I modules*	Global psychopathology	*	*	*	*
DESS	Discontinuation symptoms	*	*	*	*
IDS-C	Depressive symptoms	*	*	*	*
STAI	Anxiety trait & state	*	*	*	*
M.I.N.I. plus Suicidal	Suicide cognitions	*	*	*	*
RRS-brooding	Rumination brooding	*	*	*	*
MHC-SF	Positive mental health	*	*	*	*
FFMQ-SF	Mindfulness skills	*	*	*	*
SCS-SF	Self compassion	*	*	*	*
TIC-P	Direct and indirect costs	*	*	*	*
EQ-5D	Quality of life	*	*	*	*

Note. *Scid I modules screening, mood episodes & disorders and anxiety disorders. All questionnaires are online self-reports except SCID-I modules and IDS-C (interview) and daily calendar (pen&paper). If participants do not have internet-access paper questionnaires will be offered

#### Primary outcome measure

Our primary outcome will be full discontinuation of antidepressant medication (= 0 mg) within 6 months after starting the intervention. Use of medication will be measured with daily calendars. Patients will be invited to fill out paper calendars describing their type of antidepressant, daily milligrams used and consultations to GP or mental health assistant regarding tapering. In case of missing data in the reports, the GP will be contacted to check the GP prescription database. This latter solution is considered reliable as in the Netherlands all patients are linked to only one GP who collects all medical information for a particular patient.

#### Secondary outcome measure

- *Relapse/recurrence in depression and/or anxiety* will be measured by the modules ‘screening, mood episodes & disorders and anxiety disorders’ of the Structural Clinical Interview for DSM-IV Axis I Disorders (SCID-I), as the SCID-I for DSM-V was not available at the start of the project. Interviews will be conducted by qualified research assistants who received one full day of training to use the SCID-I. Interviews will be audio taped to allow second-rating by an independent and blind assessor in a random sample of tapes in order to prevent bias. Previous studies on inter-rater reliability of the SCID-I have reported Cronbach’s alpha values between 0.61 and 0.80 [32]
- *Withdrawal symptoms* will be assessed by means of the Discontinuation-Emergent Signs and Symptoms, DESS. This self-report will be administered to all patients using either SSRI, SNRI, TCA or other antidepressant medication. The checklist consists of 43 items and is based on an evaluation of signs and symptoms associated with discontinuation or interruption of SSRI treatment, as reported in the available literature [33]. A Dutch translation of DESS by Grootheest will be used.
- *Depressive symptoms* will be measured by the Inventory for Depressive Symptoms, IDS-C. The 30-item interview is designed to assess the severity of depressive symptoms. In this study we use the clinician rated version. The IDS-C has good psychometric qualities [34, 35].
- *Suicide cognitions* will be measured by the module Suicide Cognitions of the Mini International Neuropsychiatric Interview, MINI. The 6-item interview is designed to assess suicidal cognitions over the last month and to specify suicidal risk. In this study we use the clinician rated version [36].
- *Anxiety symptoms* will be measured by the State-Trait Anxiety Inventory, STAI [37]. A self-report measure which has been proven reliable and sensitive in the assessment of both state and trait levels of anxiety. It is a standard international measure in anxiety research and its Dutch translation has been validated [38].
- *Ruminative Brooding* will be measured by the brooding subscale of the extended version of the Ruminative Response Scale (RRS-EXT) which consists of 5 items. The authors reported adequate internal consistency *α* = .79 and test–retest stability (*α* = .62, 1 year time interval) [39].
- *Positive mental health* will be assessed with the 14-item Dutch short-form version of the Mental Health Continuum, MHC-SF [40]. The self-report questionnaire assesses emotional, psychological and social well-being. It has adequate psychometric qualities in terms of good internal consistency, (moderate) test-retest reliability, and good divergent and convergent validity.
- *Mindfulness skills* will be assessed with the Dutch short form of the Five Facet Mindfulness Questionnaire, FFMQ-SF [41]. This is a self-report inventory that specifies five mindfulness skills: observing, describing, acting with awareness, non-judging and non-reactivity. It consists of 24 items. The FFMQ has demonstrated to be a reliable and valid measure in assessing mindfulness in people with depressive symptoms.
- *Self-compassion* will be measured with the 12-item Dutch short-form version of the Self-Compassion Scale, SCS-SF [42]. The scale consists of six components: self-kindness, self-judgment, common humanity, isolation, mindfulness and over-identification. The SCS-SF has good reliability and validity.

#### Cost-effectiveness

- *Resource use* such as use of care, medication and illness related to work will be measured by means of the TIC-P. The TIC-P is a self-report inventory that consists of 38 items and measures both direct costs, i.e. care consumption of people suffering from psychiatric illness, and indirect costs, i.e. costs associated with production loss over the 12 month follow up period [43].
- To evaluate *quality of life* the EuroQol-5D, self-report version, will be used [44]. The EQ-5D uses a descriptive system of health-related quality of life states consisting of five dimensions (mobility, self-care, usual activities, pain/discomfort and anxiety/depression) each of which can take one of three responses. In addition it contains a visual analogue scale to determine Quality Adjusted Life years (QALYs). The Dutch translation will be used.

### Randomization and statistical analysis

To prevent a crossover effect between the interventions, cluster-randomization will be performed with the GP as the unit of clustering. A senior academic statistician will produce the randomization schedule based on a stratified block randomization. Stratification variables consist of region (north, central and east Netherlands) and type of practice (single versus group practice). This schedule will be send in a protected Excel-sheet to a research assistant who will allocate the GP in order of their entry to either intervention or control group. The research assistant will communicate the allocation to the researcher who will, in turn, communicate this to the GP. We are not able to keep the SCID interviewer blind to the patient’s condition. The clinical outcome data will be analysed and reported according to the CONSORT guidelines, i.e. both on an intention-to-treat and per protocol basis. With regard to missing data, sensitivity analyses will be conducted with different types of imputation. Multilevel analyses will be used to account for the hierarchical structure of the data (i.e. multiple assessments nested within patients, patients nested within GPs, and GPs nested within practices).

### Primary analysis

The primary analysis is aimed at comparing the effects of SPD and SPD + MBCT with regard to the proportion of patients who fully discontinue their antidepressant medication within 6 months. Full discontinuation is defined as using 0 mg antidepressant medication before or at the 6-month assessment. Patients who drop out before this assessment will be classified as ‘not able to discontinue’ (for the primary outcome variable). However sensitivity analyses will be conducted to examine different types of imputation. We will use a multilevel logistic regression model for repeated measures to analyze differences between the two conditions on baseline, 6 months, 9 months and 12 months follow-up measurements.

### Secondary analyses

We will use Cox regression analysis for differences in time to relapse/recurrence in depression and/or anxiety and multilevel regression analysis for all other secondary outcomes. Baseline scores of all continuous variables will be included as covariates in the respective analyses.

### Cost-effectiveness evaluation

The cost-effectiveness evaluation will be carried out from both a medical and societal perspective considering direct health related costs and societal costs following the Dutch guideline [45]. Total costs, measured with the TIC-P, will be estimated using an incremental bottom-up (micro-costing) approach, where information on each element of service used is multiplied by an appropriate unit cost and summed to provide an overall total cost [46]. We will consider four types of costs: (1) intervention costs of offering the MBCT, (2) costs stemming from health care uptake related to discontinuation, (3) patients’ and their family’s out-of-pocket costs, (4) costs stemming from productivity losses due to absenteeism related to discontinuation. The recall period will be 4 weeks at baseline, and will consist of the entire period between each of the follow-up assessments. Intervention costs will include all the costs that will contribute to the development and administration of the MBCT, for example costs of training and travel expenses. Total costs for each patient will be obtained by multiplying these data with standard costs, based on the Dutch guideline for costing research [45]. Cost as outcome are mostly skewed. In our analysis we will take skewness into account by using a GLM approach with a gamma distribution and log link. Cluster effects will be accounted for by cluster robust standard errors. Descriptive statistics on costs will be presented as well. Health-related quality of life from baseline up to 12 months will be used to calculate QALYs based on the EQ-5D using the trapezium rule. Incremental cost-effectiveness will be expressed in terms of incremental costs per QALY gained. Bootstrap simulations with 1,000 replications will be used to produce uncertainty intervals in our economic evaluation, taking into account the correlations between costs and QALYs. A cost-effectiveness acceptability curve (CEAC) will be produced to show the probabilities that either of the tested strategies is the cost-effective option, given certain values for the cost-effectiveness threshold (i.e. a willingness to pay for a QALY gained). If there are baseline differences between groups a net monetary benefit and regression based framework will be used. Uncertainty surrounding non stochastic parameters (like prices) will be explored using one way sensitivity analyses.

### Qualitative evaluation

In addition to the quantitative analysis, we also aim to conduct a qualitative study according to Consolidated criteria for reporting qualitative research, COREQ [47] . We will conduct one-on-one, in-depth interviews with a purposive sample of patients willing to discontinue their antidepressant medication about barriers and facilitators of this process.

## Discussion

Both patients and professionals seem to call for appropriate tools and information to better support the process of discontinuing antidepressant medication. In the current study we will address this gap by devising a protocol for shared decision making and the accompanying materials (i.e. information brochure, decision aid, and tapering protocol). This may serve a structured and comprehensive way to approach discontinuation.

One of the strengths of this study is its inclusive sampling strategy, allowing all patients with long-term ADM use to consider tapering and participation. Rather than a priori precluding patients with a certain level of symptomatology, for example with depressive or anxiety disorder, we used a shared decision making approach (including topics related to psychological functioning and effectiveness of antidepressant medication) to define the final RCT population. The decision to taper an antidepressant seems multifaceted, and probably does not solely depend on the level of symptoms. Another advantage of this strategy is that rather than tapering, patients and their GPs might consider switching to a different medication if that seems more appropriate.

In addition this will be the first study that will directly compare MBCT to facilitate discontinuation as an add-on to support in general practice, with support alone. This study will provide more information about the entire process of discontinuation of antidepressants in a population of long-term users in general practices, including the views and preferences of GPs and patients with regard to discontinuation, the value of using psycho-education material about tapering, and the possible value of MBCT to support the process.

## Data Availability

Not applicable.

## Abbreviations

ADM: Antidepressant medication
CBT: Cognitive–behavioural therapy
DESS: Discontinuation-Emergent Signs and Symptoms
EQ-5D: Euro-Quality of Life 5D
FFMQ: Five Facet Mindfulness Questionnaire
GP: General Practitioner
IDS: Inventory for Depressive Symptoms
MBCT: Mindfulness-Based Cognitive Therapy
MBSR: Mindfulness-based stress reduction
MHA: Mental Health Assistant
MHC: Mental Health Continuum
MINI: Mini International Neuropsychiatric Interview
RRS: Ruminative Response Scale
SCID-I: Structural Clinical Interview for DSM-IV Axis I Disorders
SCS: Self-Compassion Scale
SF: Short form
SPD: Supported Protocolized Discontinuation
STAI: State-Trait Anxiety Inventory
TIC-P: Trimbos and iMTA questionnaire on Costs associated with Psychiatric illness

## References

[1] McCarthy M (2013). Antidepressant use has doubled in rich nations in past 10 years. BMJ.

[2] Foundation of Pharmaceutical Statistics (2014). Facts and figures 2014. On pharmaceutical care in The Netherlands.

[3] Borges S, Chen YF, Laughren TP, Temple R, Patel HD, David PA, Mathis M, Unger E, Yang P, Khin NA (2014). Review of maintenance trials for major depressive disorder: a 25-year perspective from the US Food and Drug Administration. J Clin Psychiatry.

[4] Bet PM, Hugtenburg JG, Penninx BW, Hoogendijk WJ (2013). Side effects of antidepressants during long-term use in a naturalistic setting. Eur Neuropsychopharmacol.

[5] Ferguson JM (2001). SSRI antidepressant medications: adverse effects and tolerability. Prim Care Companion J Clin Psychiatry.

[6] Coupland C, Dhiman P, Morriss R, Arthur A, Barton G, Hippisley-Cox J (2011). Antidepressant use and risk of adverse outcomes in older people: population based cohort study. BMJ.

[7] National Institute for Health and Care Excellence (2009). Depression in adults: recognition and management. CG90.

[8] Baldessarini RJ, Tondo L, Ghiani C, Lepri B (2010). Illness risk following rapid versus gradual discontinuation of antidepressants. Am J Psychiatry.

[9] Moore M, Yuen HM, Dunn N, Mullee MA, Maskell J, Kendrick T (2009). Explaining the rise in antidepressant prescribing: a descriptive study using the general practice research database. BMJ.

[10] Johnson CF, Macdonald HJ, Atkinson P, Buchanan AI, Downes N, Dougall N (2012). Reviewing long-term antidepressants can reduce drug burden: a prospective observational cohort study. Br J Gen Pract.

[11] Petty DR, House A, Knapp P, Raynor T, Zermansky A (2006). Prevalence, duration and indications for prescribing of antidepressants in primary care. Age Ageing.

[12] Mojtabai R, Olfson M (2014). National trends in long-term use of antidepressant medications: results from the U.S. National Health and nutrition examination survey. J Clin Psychiatry.

[13] Meijer WE, Heerdink E, Leufkens HG, Herings RM, Egberts AC, Nolen WA (2004). Incidence and determinants of long-term use of antidepressants. Eur J Clin Pharmacol.

[14] Verbeek-Heida PM, Mathot EF (2006). Better safe than sorry--why patients prefer to stop using selective serotonin reuptake inhibitor (SSRI) antidepressants but are afraid to do so: results of a qualitative study. Chronic Ill.

[15] Leydon GM, Rodgers L, Kendrick T (2007). A qualitative study of patient views on discontinuing long-term selective serotonin reuptake inhibitors. Fam Pract.

[16] Fava GA, Gatti A, Belaise C, Guidi J, Offidani E (2015). Withdrawal symptoms after selective serotonin reuptake inhibitor discontinuation: a systematic review. Psychother Psychosom.

[17] Eveleigh R (2015). Inappropriate long-term antidepressant use in primary care: a challenge to change.

[18] Guidi J, Tomba E, Fava GA (2016). The sequential integration of pharmacotherapy and psychotherapy in the treatment of major depressive disorder: a meta-analysis of the sequential model and a critical review of the literature. Am J Psychiatry.

[19] Segal ZV, Williams JMG, Teasdale JD. Mindfulness-Based Cognitive Therapy for Depression. 2nd ed: Guilford Press; 2012.

[20] Kabat-Zinn J. Full catastrophe living: using the wisdom of your body and mind to face stress, pain and illness. New York: Bantam; 2013.

[21] Beck AT, Rush AJ, Shaw BF, Emery G. Cognitive therapy of depression. New York: Guilford press; 1979.

[22] Kuyken W, Warren FC, Taylor RS, Whalley B, Crane C, Bondolfi G, Hayes R, Huijbers M, Ma H, Schweizer S (2016). Efficacy of mindfulness-based cognitive therapy in prevention of depressive relapse: an individual patient data meta-analysis from randomized trials. JAMA Psychiatry.

[23] Kuyken W, Byford S, Taylor RS, Watkins E, Holden E, White K, Barrett B, Byng R, Evans A, Mullan E (2008). Mindfulness-based cognitive therapy to prevent relapse in recurrent depression. J Consult Clin Psychol.

[24] Kuyken W, Hayes R, Barrett B, Byng R, Dalgleish T, Kessler D, Lewis G, Watkins E, Brejcha C, Cardy J (2015). Effectiveness and cost-effectiveness of mindfulness-based cognitive therapy compared with maintenance antidepressant treatment in the prevention of depressive relapse or recurrence (PREVENT): a randomised controlled trial. Lancet (London, England).

[25] Huijbers MJ, Spinhoven P, Spijker J, Ruhe HG, van Schaik DJ, van Oppen P, Nolen WA, Ormel J, Kuyken W, van der Wilt GJ (2016). Discontinuation of antidepressant medication after mindfulness-based cognitive therapy for recurrent depression: randomised controlled non-inferiority trial. Br J Psychiatry.

[26] Campbell MK, Mollison J, Steen N, Grimshaw JM, Eccles M (2000). Analysis of cluster randomized trials in primary care: a practical approach. Fam Pract.

[27] Kroenke K, Spitzer RL, Williams JB (2001). The PHQ-9: validity of a brief depression severity measure. J Gen Intern Med.

[28] Spitzer RL, Kroenke K, Williams JB, Löwe B (2006). A brief measure for assessing generalized anxiety disorder: the GAD-7. Arch Intern Med.

[29] First MB, Gibbon M, Spitzer RL, Williams JB (1996). User’s guide for the structured clinical interview for DSM-IV axis I disorders—research version.

[30] Teasdale JD, Williams JMG, Segal ZV. The mindful way workbook: an 8-week program to free yourself from depression and emotional distress. New York: Guilford publications; 2013.

[31] Crane RS, Eames C, Kuyken W, Hastings RP, Williams JM, Bartley T, Evans A, Silverton S, Soulsby JG, Surawy C (2013). Development and validation of the mindfulness-based interventions - teaching assessment criteria (MBI:TAC). Assessment.

[32] Lobbestael J, Leurgans M, Arntz A (2011). Inter-rater reliability of the structured clinical interview for DSM-IV Axis I disorders (SCID I) and Axis II disorders (SCID II). Clin Psychol Psychother.

[33] Rosenbaum JF, Fava M, Hoog SL, Ascroft RC, Krebs WB (1998). Selective serotonin reuptake inhibitor discontinuation syndrome: a randomized clinical trial. Biol Psychiatry.

[34] Rush AJ, Gullion CM, Basco MR, Jarrett RB, Trivedi MH (1996). The inventory of depressive symptomatology (IDS): psychometric properties. Psychol Med.

[35] Trivedi MH, Rush AJ, Ibrahim HM, Carmody TJ, Biggs MM, Suppes T, Crismon ML, Shores-Wilson K, Toprac MG, Dennehy EB (2004). The inventory of depressive symptomatology, clinician rating (IDS-C) and self-report (IDS-SR), and the quick inventory of depressive symptomatology, clinician rating (QIDS-C) and self-report (QIDS-SR) in public sector patients with mood disorders: a psychometric evaluation. Psychol Med.

[36] Sheehan DV, Lecrubier Y, Sheehan KH, Amorim P, Janavs J, Weiller E, Hergueta T, Baker R, Dunbar GC (1998). The Mini-International Neuropsychiatric Interview (M.I.N.I.): the development and validation of a structured diagnostic psychiatric interview for DSM-IV and ICD-10. J Clin Psychiatry.

[37] Spielberger CD (1985). Assessment of state and trait anxiety: conceptual and methodological issues. South Psychol.

[38] Van der Ploeg H (1980). Validity of the Zelf-Beoordelings-Vragenlijst (a Dutch version of the Spielberger state-trait anxiety inventory). Nederlands Tijdschrift Psychol Grensgebieden.

[39] Treynor W, Gonzalez R, Nolen-Hoeksema S (2003). Rumination reconsidered: a psychometric analysis. Cogn Ther Res.

[40] Lamers SM, Westerhof GJ, Bohlmeijer ET, ten Klooster PM, Keyes CL (2011). Evaluating the psychometric properties of the mental health continuum-short form (MHC-SF). J Clin Psychol.

[41] Bohlmeijer E, ten Klooster PM, Fledderus M, Veehof M, Baer R (2011). Psychometric properties of the five facet mindfulness questionnaire in depressed adults and development of a short form. Assessment.

[42] Raes F, Pommier E, Neff KD, Van Gucht D (2011). Construction and factorial validation of a short form of the self-compassion scale. Clin Psychol Psychother.

[43] Hakkaart-van Roijen L, Van Straten A, Donker M, Tiemens B (2002). Trimbos/iMTA questionnaire for costs associated with psychiatric illness (TIC-P).

[44] The EuroQol Group (1990). EuroQol--a new facility for the measurement of health-related quality of life. Health Policy (Amsterdam, Netherlands).

[45] Hakkaart-van Roijen L, Van der Linden N, Bouwmans C, Kanters T, Tan SS (2015). Kostenhandleiding. Methodologie van kostenonderzoek en referentieprijzen voor economische evaluaties in de gezondheidszorg In opdracht van Zorginstituut Nederland Geactualiseerde versie.

[46] Drummond M, Manca A, Sculpher M (2005). Increasing the generalizability of economic evaluations: recommendations for the design, analysis, and reporting of studies. Int J Technol Assess Health Care.

[47] Tong A, Sainsbury P, Craig J (2007). Consolidated criteria for reporting qualitative research (COREQ): a 32-item checklist for interviews and focus groups. Int J Qual Health Care.

